# Compensatory angiogenesis and tumor refractoriness

**DOI:** 10.1038/oncsis.2015.14

**Published:** 2015-06-01

**Authors:** R N Gacche

**Affiliations:** 1 Tumor Biology Laboratory, School of Life Sciences, Swami Ramanand Teerth Marathwada University, Nanded, India

## Abstract

Since the establishment of tumor angiogenesis as a therapeutic target, an excitement in developing the anti-angiogenic agents was resulted in tailoring a humanized monoclonal antibody (Bevacizumab) against vascular endothelial growth factor (VEGF): a key factor in recruiting angiogenesis. The past three decades' research in the area of angiogenesis also invented a series of novel and effective anti-angiogenic agents targeting the VEGF signaling axis. Despite the demonstrable clinical benefits of anti-angiogenic therapy, the preclinical and clinical data of the current therapeutic settings clearly indicate the transient efficacy, restoration of tumor progression and aggressive recurrence of tumor invasion after the withdrawal of anti-angiogenic therapy. Therefore, the impact of this therapeutic regime on improving overall survival of patients has been disappointing in clinic. The recent advances in pathophysiology of tumor angiogenesis and related molecular and cellular underpinnings attributed the conspiracy of compensatory angiogenic pathways in conferring evasive and intrinsic tumor resistance to anti-angiogenic agents. The understandings of how these pathways functionally cross-talk for sustaining tumor angiogenesis during VEGF blockade is essential and perhaps may act as a basic prerequisite for designing novel therapeutic strategies to combat the growing arrogance of tumors toward anti-angiogenic agents. The present review offers a discourse on major compensatory angiogenic pathways operating at cellular and molecular levels and their attributes with resistance to anti-angiogenic agents along with strategic opinions on future setting in targeting tumor angiogenesis.

## Introduction

Angiogenesis is a physiological process of formation of new capillaries on pre-existing vessels. The recent literature in the area of development of blood vessels is impressive in understanding the dynamics and complexities of vasculogenesis/angiogenesis.^1^ The process of angiogenesis appears to be fundamental for retrieving continues supply of oxygen and nutrients. The sprouting of new blood vessel initiates with dissolution of vascular basal membrane, increase in vascular permeability and degradation of extracellular matrix, followed by endothelial cell (EC) migration, invasion, proliferation and tube formation. The angiogenic switch refers to a consortium of several regulatory factors, which regulates angiogenesis by maintaining a strict balance between activators and inhibitors in normal physiological angiogenesis. The pathological angiogenesis is mostly a hallmark of cancer, wherein the developed vasculature is more complex, abnormal, leaky and torturous.^2^

In 1971, Judah Folkman, a pioneer researcher in tumor angiogenesis, first highlighted the significance of vasculature for the growth and proliferation of solid tumors. He demonstrated that if a tumor is deprived from generating its own blood supply, it would not grow more than 1–2 mm in size or it may wither and die. Since the opening of this research window, the preclinical and clinical data started accumulating in a logarithmic manner, with a clear intention of inhibiting the tumor angiogenesis.^2^ Till date, 10 anti-angiogenic agents have been approved by the US Food and Drug Administration: bevacizumab and ziv-aflibercept as anti-vascular endothelial growth factor (VEGF) agents, whereas sorafenib, sunitinib, pazopanib, axitinib, cabozantinib and regorafenib are approved as small-molecule RTK (receptor tyrosine kinase) inhibitors.^3^

Besides several appreciation reports describing the efficacy of anti-angiogenic agents in extending the survival of cancer patients by few months,^4^ the patients' benefit from the treatment is not satisfactory and is rather disappointing because of transient and modest performance of the anti-angiogenic agents in the clinic, off-target toxicities and intrinsic refractoriness.^2^ Perhaps more serious is the aggressive invasion and expedite metastasis of tumors after withdrawal of anti-angiogenic drugs.^5^ Besides numerous clinical trials in progress and over dozens of molecules being engineered against VEGF/VEGF receptor (VEGFR) and non-VEGF pathways,^4, 6^ the entire situation warrants the understanding of molecular underpinnings and loopholes supporting the bypass angiogenic mechanisms ensuring tumor progression and metastasis even after the treatment with effective anti-angiogenic agents.

## Tumors employ multiple compensatory pro-angiogenic factors and signaling pathways in anti-VEGF environment

**Figure 1:**
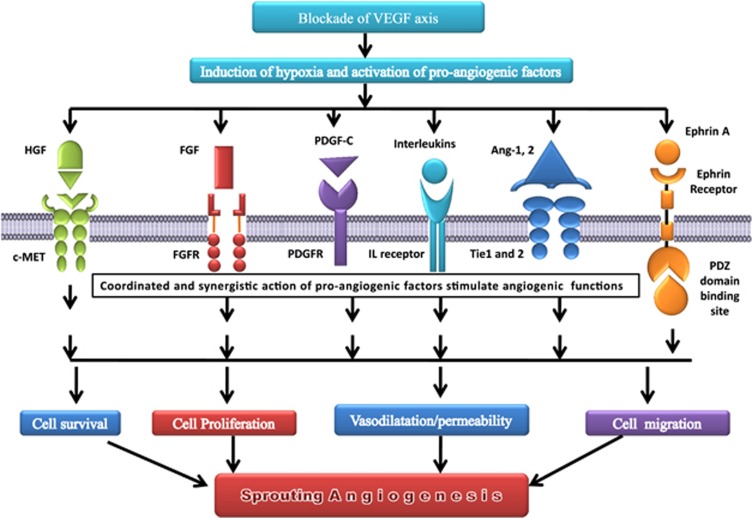
Role of pro-angiogenic factors in driving compensatory angiogenesis during blockade of VEGF axis.

**Figure 2:**
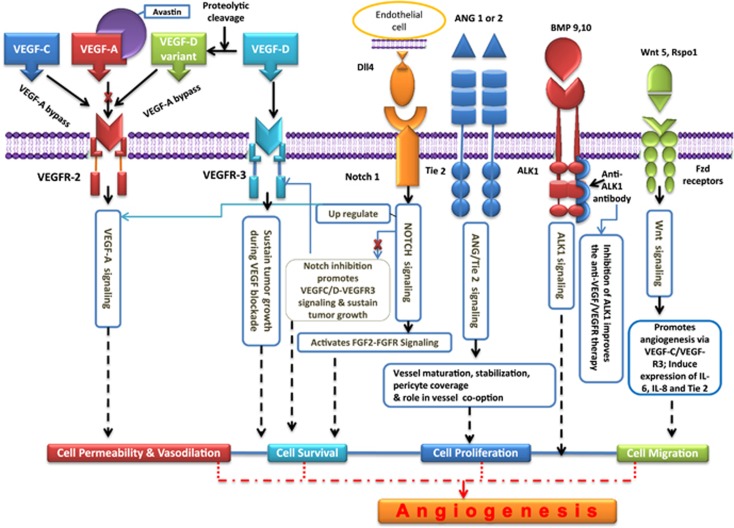
Compensatory angiogenic pathways.

In a broader sense, the multiple compensatory angiogenic factors/signaling pathways that tumors employ during anti-VEGF stress (Figures 1 and 2,) can be conveniently categorized as VEGF-dependent pathways, VEGF-independent signaling, mechnisms involving myeloid/stromal/tumor cell interactions and angiogenesis-independent vascular remodeling processes such as vessel cooption, intussusceptions and vascular mimicry.

### VEGF-dependent compensatory angiogenic mechanisms

The VEGF axis-dependent alteration pathways are initiated by the subtypes of VEGF such as VEGF-B, VEGF-C, VEGF-D and placenta growth factor (PIGF) (Figure 2). VEGF-B is observed to be upregulated in multiple malignancies having anticipatory role in supporting tumor cell migration.^7^ Ziv-aflibercept (Zaltrap, VEGF-Trap) is an antibody linked to IgG1 backbone and possesses VEGFR1 and VEGFR2 as extracellular domains. Its binding preference is not only with VEGF-A but also with VEGF-B and PlGF. The role of VEGF-C and VEGF-D is primarily implicated in lympangiogenesis.^8^ It has been reported that a VEGF-C fragment generated through proteolytic cleavage has binding affinity with VEGFR-3 and perhaps could be an alternative bypass mechanism and a potential cause of resistance to anti-VEGF-A modalities.^9^ NRP-1 (VEGF receptor) not only binds to VEGF-A but also binds to PlGF. Moreover, NRP-2 interacts with VEGFR-2 and may enhance angiogenic signaling. Apart from VEGF-A, NRP-2 also binds with VEGF-C and PlGF, thereby having a significant role in angiogenesis.^10^ Clinical evidences also strengthen the possibility of involvement of PlGF in angiogenic rescue task, as upregulated levels of PlGF were detected in patients treated with anti-VEGFR therapy.^11^

### VEGF-independent compensatory pro-angiogenic factors and signaling mechanisms

Clinical and experimental settings have identified several angiogenic growth factors having a key role in anti-VEGF escape mechanisms (Figure 1). Some of the key angiogenic factors involved in this puzzle of compensatory angiogenic signaling (Figure 2) includes fibroblast growth factors 1 and 2 (FGF1 and FGF2, respectively),^12^ hepatocyte growth factor/cMet pathway,^13^ angiopoietins,^14^ Delta4–Notch signaling pathway,^15^, platelet-derived growth factor (PDGF)-C,^16^ interleukins,^17^ Ephrins,^18^ ALK1 signaling^19^ and Wnt signaling.^20^ FGF signaling has been considered as one of the major culprit for adaptive tumor resistance to VEGF-targeted inhibitors.^12, 21^ In fact, binding of FGF receptors to FGF ligands causes activation of several signaling pathways such as phospholipase Cγ–protein kinase C, phosphatidylinositol 3-kinase (PI3K-AKT-mTOR), JAK/STAT (janus kinase-signal transducer and activator of transcription) and MAPK (mitogen-activated protein kinase: RAS/RAF/MAPK and RAS/MAPK/ERK).^22^ The signaling and cross-talk of these pathways (Figure 2) are involved in regulation of myriad of physiological processes including angiogenesis even after treating the tumors with VEGF-blocking agents.^23^ A clear evidence of evasive resistance mediated by FGF-dependent revascularization was observed in patients with recurrent glioblastoma taking treatment of cediranib (Recentin, Astra Zeneca, London, UK), a VEGFR inhibitor. Moreover, inhibition of FGF/VEGF using brivanib was found to be effective in treating mouse pancreatic neuroendocrine tumors demonstrating adaptive/evasive resistance to VEGF inhibition.^24^ Plethora of preclinical and clinical findings has highlighted the significance of Delta4–Notch signalling as one of the pathways mediating the tumor refractoriness to anti-VEGF therapy.^9, 15, 25, 26^ Inhibition of Delta4 leads to deregulation of angiogenesis, thereby resulting in excessive but non-functional vasculature, which can be conveniently used as an effective strategy for paralyzing the tumor growth that are relatively resistant to anti-VEGF therapeutic regime and for improving the therapeutic index of anti-VEGF agents by combining it with Delta4 inhibitors.^26^

In an investigation designed to understand the modes of tumor resistance to sunitinib, the HGF/c-Met pathway was identified as a culprit for anti-VEGF rescue mechanism.^27^ In clinical trial studies, in hepatocellular carcinoma (HCC) patients who were treated with VEGFR inhibitor, sorafenib, the progression-free survival was strongly correlated with low serum HGF levels as compared with patients with high serum HGF levels having progressive development of the disease.^28^ In recent cell coculture studies, a stromal cell (HSC-LX2) derived from the microenvironment of HCC was able to induce sorafenib resistance in HCC cells (Huh7) through multiple pathways including HGF/c-Met/Akt and Jak2/Stat3 (Chen *et al.*^29^).

The vessel normalization hypothesis claims that anti-VEGF agents, especially VEGFR2 blockers, normalize the vessels by proper covering of pericytes over the leaky vessels. This is a kind of vessel improvement and perhaps may act as an angiogenic escape mechanism. This proposed mechanism appears to be mediated by ANG1/Tie2 signaling.^30^ In case of MMTV-PyMT mammary carcinomas, the higher levels of ANG2 were associated with worse response to anti-angiogenic therapy containing bevacizumab. Nevertheless, there was lack of synergistic anti-angiogenic or antitumor functions arising out of ANG2/VEGFR2 blockade.^31^ During VEGF arrest, administration of BowANG1 (an engineered construct that induces phosphorylation of Tie-2 in cultured ECs successfully prevented tumor regression by VEGF trap.^32^ The major concerns of PDGFs in anti-VEGF rescue operations are largely associated with its role in activating pro-angiogenic stromal/perivascular cells conferring tumor resistance.^9^ In mouse lymphoma tumor model studies, the anti-VEGF-resistant tumor cells overexpressed PDGF-C mRNA and activated the adjacent tumor-associated fibroblasts (TAFs) to secrete PDGF-C, which in turn triggered tumor angiogenesis.^16^

Several members of interleukins (ILs) such as IL-1, IL-8, IL-12 (Vasudev and Reynolds)^6^ and, more recently, IL-17 (Chung *et al.*^33^) have been implicated in tumor refractoriness to anti-VEGF agents. A clear involvement of IL-8 has been reported in anti-VEGF tumor resistance in sunitinib-treated renal cell carcinoma.^17^ Chung *et al.*^33^ established a new link of IL-17-driven and stromal cell-mediated signaling that confers resistance to anti-VEGF agents. Chung *et al.*^33^ also showed that IL-17 signaling cascade mobilizes the granulocyte colony-stimulating factor (CSF)-dependent recruitment of CD11b^+^Gr1^+^ immature myeloid cells, which acts as major driving force for anti-VEGF tumor refractoriness.^34^ Mostly, the role of EphA2/EphrinA1 signaling has been attributed with compensatory angiogenesis and concerned tumor resistance. When the functions of VEGFR1 and R2 were blocked using antibodies, the resistance toward VEGFR2 blockade was observed in the form of revascularization and regrowth of tumors. The resistance to VEGF blockade was strongly correlated with hypoxia-induced upregulation of Ephrin A1 and other compensatory pro-angiogenic factors.^35^ In pancreatic tumors, overexpression of Eph A2 and Ephrin A1 was observed in VEGF-treated tumors and furthermore the elevated levels were correlated with tumor refractoriness.^35, 36^

Suzuki *et al.*^37^ have demonstrated the pro-angiogenic effects of ALK1/BMP9 signaling, which includes proliferation of ECs, promotion of tumor angiogenesis in matrigel plug vascularization in a xenograft model of pancreatic cancer. In melanoma xenografts with upregulated human VEGF-A and resistance to RTK inhibitor, the combined treatment of bevacizumab in conjunction with PF-03446962 (a human IgG2 monoclonal anti-ALK1 antibody) significantly improved the efficacy of VEGF/VEGFR targeting agents, thus giving a clue of involvement of ALK1 signaling in bevacizumab resistance and thereby in promoting angiogenesis under VEGF blockade.^38^ The combination therapy of VEGFR tyrosine kinase inhibitor and ALK1 inhibitor (ALK1-Fc) has also been assessed in VHL-deficient RCC murine xenograft models, where a clear evidence of resistance towards anti-VEGFR agents was observed.^19^ In zebrafish model studies, Rspo1/Wnt-mediated signaling promotes angiogenesis via VEGF-C/VEGF-R3, but not involving VEGF-A. The authors hope that the novel Rspo-Wnt-VegfC-Vegfr3 pathway may have an important role as bypass angiogenic pathway during tumor angiogenesis.^20^

### Stromal/tumor cells recruited pro-angiogenic conspiracy and tumor refractoriness

#### BMDCs: a reservoir of vascular progenitor cells

**Figure 3:**
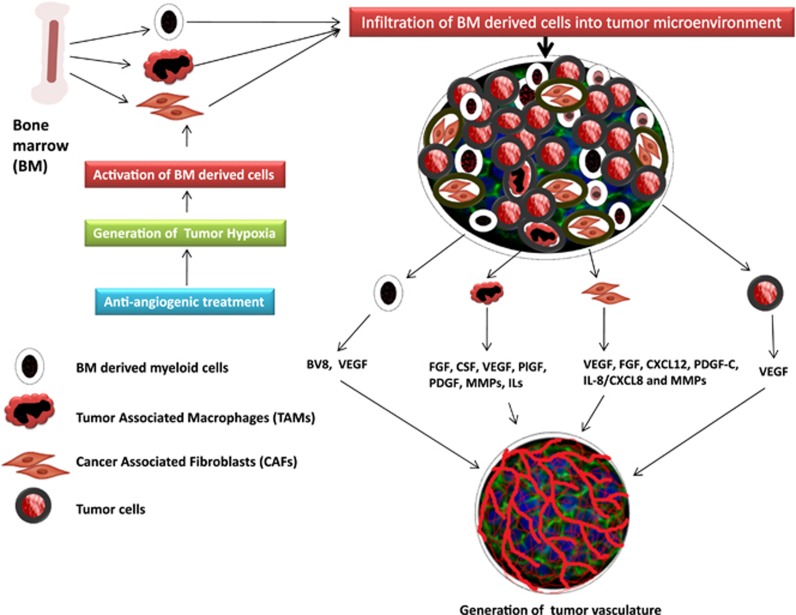
Role of stromal and tumor cells in compensatory angiogenesis.

Bone marrow-derived cells (BMDCs) constitute a major reservoir of vascular progenitor and vascular modulating cells, which infiltrate into stroma of tumors and have an important role in tumor progression including promotion of tumor angiogenesis independent of VEGF.^9, 39^ The most prominent cell populations of the BMDCs contributing to the tumor angiogenesis and progression (Figure 3) includes a lineage of GR1^+^CD11b^+^ myeloid progenitors, CD11b^+^CD13^+^ myeloid cells, CXCR4^+^VEGFR1^+^ hemangiocytes, tumor-associated macrophages (TAMs) expressing CD11b^+^F4/80^+^, Tie2-expressing monocytes, (PDGFR)^+^ pericyte progenitors, a population of CD45^+^CD11b^+^ myeloid cells, tumor-infiltrated mast cells and neutrophils, and vascular endothelial-cadherin^+^ CD45^+^ vascular leukocytes and so on.^39, 40^ Besides these prominent pro-angiogenic BMDCs, several other lineages of myeloid cells involved in tumor progression and metastasis are extensively reviewed elsewhere.^41, 42^ An investigation probing the mechanisms of tumor resistance toward the anti-angiogenic agents, a bone marrow-derived (BMD) CD11b^+^Gr1^+^ lineage encountered in anti-VEGF rescue mechanism, has been well documented.^43^ The CD11b^+^Gr1^+^-driven angiogenesis was kind of co-ordinated consequence of a granulocyte–macrophage colony-stimulating factor, SDF-1α, placenta growth factor, granulocyte CSF and the granulocyte CSF-induced Bv8 (Bombina variagata peptide 8)-secreted protein.^43^ In brief, the CD11b^+^Gr1^+^-mediated angiogenesis is partly driven by the Bv8-dependent pathway, which is upregulated by granulocyte CSF that escapes VEGF and renders tumors more resistant to anti-VEGF agents.^44^

BMD TAMs have been considered as a primary cause of poor prognosis and resistance to anti-VEGF agents.^45^ TAM-mediated regulation of angiogenesis has been extensively investigated in animal tumor model studies.^46, 47^ PLGF was overexpressed on anti-VEGFR treatment; PLGF-mediated recruitment of pro-angiogenic and resistance-conferring TAMs might be a plausible mechanism for compensatory angiogenesis and tumor resistance.^48^ Nevertheless, the treatment of sorafenib in HCC tumors was also associated with elevated levels of CSF-1, SDF-1α and VEGF, which *per se* are chemokines for inviting macrophages.^46^ The finding of the HCC tumor model studies pinpoints the involvement of TAMs in tumor angiogenesis and progression under the treatment of sorafenib, which inhibits the VEGFR2, PDGF receptor and Raf kinases.

The current state-of-the-art literature cited elsewhere focus on several dimensions of TAF-mediated cues in tumor invasion, progression, angiogenesis and metastasis.^49, 50^ The stroma and the invasive front of a tumor are usually occupied by clad of TAFs in different types of cancers such as lung, prostrate, breast, pancreas and colon.^49^ In particular, TAF-mediated conspiracy of driving angiogenesis under VEGF blockade is considered as a prominent cause of anti-VEGF tumor refractoriness (reviewed in Crawford and Ferrara,^9^ Ferrara^21^ and Öhlund *et al.*^51^). Crawford *et al.*^16^ demonstrated that TAFs derived from resistant tumors sustained tumor growth and angiogenesis even after arresting the functions of VEGF. Further, it was found that TAF generated PDGF-C was a key factor in sustaining angiogenesis and tumor growth under anti-VEGF environment. The involvement of TAF-generated PDGF-C in tumor refractoriness was confirmed by arresting its functions using neutralizing antibody, which subsequently ameliorated the TAF-resistant induced angiogenesis and drastically delayed the growth of resistant tumors.^16^ di Tomaso *et al.*^52^ also demonstrated that the stroma-generated PDGF-C overexpression was associated with resistance to anti-VEGF treatment in glioblastoma tumor model studies.^52^

#### Pericytes and CSCs sustain tumors in anti-VEGF environment

Pericytes (*peri*: around; *cyte*: cell) are the contractile perivascular cells that wrap around the newly formed blood capillaries. It is now a well-established fact that pericytes have a significant role in pro-angiogenic signaling, especially in vascular morphogenesis, and have direct regulatory control over EC proliferation or quiescence.^53^ Under hypoxic environment, the upregulated expression of VEGF-A in mature PCs recruit them to newly formed vessels.^5^ Pericytes also protect ECs through upregulation of Bcl-w protein, which protects ECs from apoptosis.^54^. Of note, anti-angiogenic agents not only prune the neovasculature but also significantly induce apoptosis in ECs.^54^ Autocrine VEGF-A signaling in ECs in association with PCs offers an understanding of how PCs protects ECs under anti-angiogenic environment and recur angiogenesis after the relapse of anti-angiogenic therapy. Upregulation of PDGF-B in tumor cells has profound effect on increasing the PC coverage^55^ and the reports state that PCs can protect ECs from VEGF withdrawal by activating compensatory pro-angiogenic signaling, especially PDGF receptor-medited angiogenic pathway in anti-VEGF therapy.^55^ In the current mainstream of tumor resistance, now it has been widely accepted that the anti-angiogenic drugs mainly prune ECs lacking PC coverage, while demonstrating limited efficacy on the PCs embarrassed mature vessels.^56^ Interestingly, low-to-moderate doses and transient treatment duration of anti-angiogenic agents have been shown to improve the efficacy of anti-angiogenic agents by a process of vessel normalization.^3^. Although the pro-angiogenic role of PCs is evolving, interestinlgy PCs derived from infantile hemangioma tumor of human demonstrated impressive pro-angiogenic activities and, of note, pro-angiogenic activity of these PCs was many fold higher than the retinal PCs.^56^

In the mainstream of cancer treatment, tumor heterogeneity has seeded several enquiries and imposed challenges in relation to its causes and consequences, concern with improving the drug efficacy and challenge of ameliorating the emerging drug resistance.^57^ Cancer stem cells (CSCs) represent a heterogenous subpopulation of cancer cells that retains the potential of self-renewal, which leads to malignant progeny. Nevertheless, CSCs have also been identified as an emerging major driving force that governs tumor recurrence and confer resistance to anti-cancer agents.^58^ Putative CSCs have been reported in several tumor types including brain, melanoma, colorectal, breast, hepatic, head and neck, and prostate cancer.^59^ Several dimensions of angiogenic functions of CSCs have been recently reviewed elsewhere.^59, 60^ The reason for suspecting CSCs in alternate angiogenesis and tumor resistance lies in their capabilities to produce much higher levels of VEGF in both anoxic and hypoxic environment than non-CSC population,^60^ which perhaps might be boosting strong angiogenic cues after the relapse of VEGF blockade. The report describes that the treatment of sunitinib and bevacizumab increases the number of breast CSCs by upregulation of hypoxia-inducible factor (HIF)-1α and through the activation of Wnt pathway via Akt/β-catenin signaling.^61^

## Hypoxia fuels pro-angiogenic stromal/tumor cells

Tumor angiogenesis and hypoxia have been identified as hallmarks of solid tumors. HIF-1α mediates the transcription of genes involved in angiogenesis, oxygen consumption, migration and invasion of cancer cells.^62^ It has been described that anti-angiogenic agents induce ‘vascular regression,' which leads to increase in intratumor hypoxia and selection of more invasive metastatic clones of the cancer cells that are resistant to therapy including anti-angiogenic agents.^63^ Nevertheless, targeting hypoxia is being prioritized for overcoming the disappointing performance of anti-angiogenic agents in the clinic.^62^

A vast body of literature describes the role of hypoxia in activating and upregulating the compensatory angiogenic factors/pathways and infiltrating different BMD pro-angiogenic cells including circulating endothelial progenitors and CSCs in tumor micro-environment.^64^. The most dramatic effect of hypoxia has been shown in recruiting BMDCs in TME, which later have an important role in compensatory angiogenesis.^64, 65^ Blocking the functions of HIF impaired the mobilization of BMD pro-angiogenic cells such as VEGFR2^+^CD34^+^, VEGFR2^+^CD117^+^ and CXCR4^+^ Sca1^+^ into the circulation and inhibition of tumor angiogenesis, thereby adversely affecting the tumor growth.^66^ Knockdown of Phd2 (a negative regulator of HIF-1α) in human colon cancer resulted in increasing the number of pro-angiogenic CD11b^+^ tumor-associated myeloid cells and promotion of vascularization.^67^ TAMs have been shown to preferentially accumulate in the hypoxic and necrotic regions in a variety of solid tumor cancers of humans such as breast, endometrium, ovary, bladder, colon and the oral cavity (reviewed in Quail and Joyce^42^). CSCs preferentially reside in hypoxic niches; moreover, they have upregulated levels of HIFs.^68^ The hypoxia induced by anti-angiogenic agents also mobilizes and increases the number of CSCs in breast cancer.^61^ Hu *et al.*^69^ have demonstrated that hypoxia-induced autophagy triggers tumor cell survival and adaptive resistance to anti-angiogenic therapy in the glioblastoma. Targeting the hypoxic microenvironment might serve effective anti-angiogenic combination therapies for combating the emerging resistance towards anti-angiogenic therapy.^64^

## Alternative mechanisms of angiogenesis-independent tumor vasculature: an oasis during anti-VEGF stress

**Figure 4:**
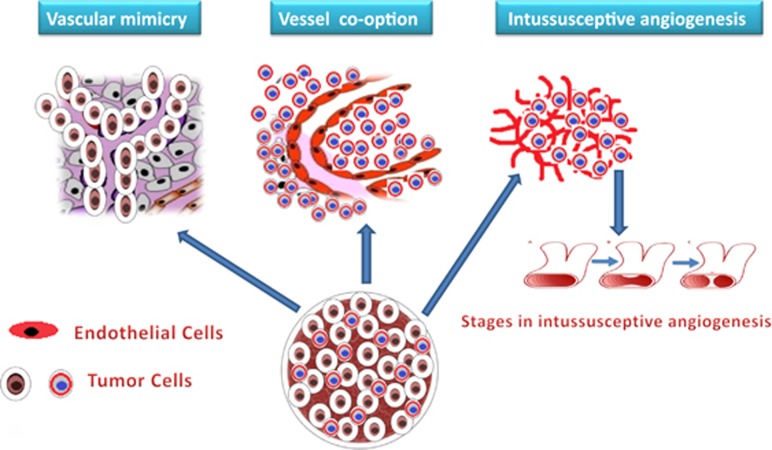
Angiogenesis-independent vascular remodeling mechanisms.

The requirement of classic angiogenesis was previously thought to be a basic prerequisite for tumor progession and metastasis. Perhaps this assumption turned out as an illusion, as now sufficient evidence states that tumors can sustain their growth through various angiogenesis-independent mechanisms such as vessel cooption, vessel remodeling through intussusception, vascular mimicry (Figure 4) and other processes such as postnatal vasculogenesis, glomeruloid and looping angiogenesis.^70^ In vessel cooption, the tumor cells embrace the local blood vessels of the host during the invasion of tumors in surrounding host tissue and migrate along the vessels of the host organs.^70, 71^ Vessel cooption is more frequently observed in cancers of densely vascularized organs such as the brain, lung and liver, wherein the primary tumor cells including metastases coopt with the adjacent existing quiescent blood vessels of the host tissue.^72^ Holash *et al.*^70^ proved that the implantation of C6 glioma cells in rat brain leads to formation of small vascularized tumors independent of angiogenesis. Kunkel *et al.*^73^ demonstrated that the systemic treatment with DC101, monoclonal antibody against VEGFR-2, increased cooption of quiescent cerebral vessels with residual tumors, which was further observed to have central cores of coopted vessels. The treatment of anti-angiogenic ZD6474 agent with brain metastases of cerebral melanoma was found to be associated with marked increase in vessel cooption.^72^ Interestingly, the coopted vessels respond differentially toward anti-angiogenic agents among different tumors. For example, liver metastases from breast cancer are more depended on cooption of the liver vasculature than that of colorectal origin.^74^

The intussusceptive microvascular growth (IMG) also called splitting angiogenesis is a novel mode of vessel generation and vascular remodeling that might be acting as an anti-VEGF rescue meditor. IMG involves formation of two new vessels through fission of the pre-existing capillary plexus without sprouting.^75^ IMG, which happens to be an adaptive response to stress and hypoxia, is observed in several human tumors such as melanoma, colon, mammary carcinomas, B-cell non-Hodgkin's lymphoma and glioblastoma (reviewed in Patan *et al.*^76^). IMG has been identified as an important mechanism of rapid vascular remodeling in colon adenocarcinoma xenograft that contributes for intermittent blood flow in tumors. ^76^ Treatment of mammary carcinoma allograft with VEGF tyrosine kinase inhibitor (PTK787/ZK222854) resulted in the development of extensive IMG in post-relapse period.^77^ In Lewis lung carcinoma and RIP-Tag 2 model studies, clear evidence of IMG and rapid revascularization was observed in tumors recovered after the treatment with VEGFR inhibitors.^78^ Perhaps, tumors might prefer IMG during anti-angiogenic exposure, as it is faster, and thermodynamically and metabolically more feasible as compared with sprouting angiogenesis.^76, 78^

Maniotis *et al.*^79^ unraveled a tumor supporting crucial mechanism of generation of endothelium-independent vascular network called vasculogenic mimicry (VM). The authors demonstrated that the aggressive human melanoma cells mimic the functions of ECs and generate vascular tunnel-like phenotype that is foolproof in carrying red blood cells and plasma without involvement of ECs.^79^ VM has been reported in different human malignant tumours such as breast, melanoma, bladder, kidney, gliomas, glioblastomas, prostrate, ovarian, lung, sarcomas, cell renal cell carcinoma and astrocytoma.^80^ The imbroglio of VM is an amalgamation of three distinct elements, the primary one is the plasticity of aggressive tumor cells, followed by remodeling of the ECM and finally establishing the connections of the VM channels with the existing network of host microvessel system.^81^ VM is critically regulated by several signaling pathways associated with embryonic/stem cell (Nodal and Notch4), vascular (VE-cadherin, VEGFR1, EphA2) and hypoxia-related HIF and Twist1 (Qiao *et al.*^82^). Clinical evidence of occurrence of VM in patients is strongly correlated with high risk of metastasis, poor prognosis, cancer recurrence and worse survival for patients of variety of cancers.^81^ Previous report entails the resistance of VM to anti-angiogenic inhibitors such as endostatin and TPN-470 in B16F10 murine melanoma model, as well as in melanoma tumor cells.^83^ It is interesting to note that hypoxia induces VM and, of note, CSCs, which possess tumor recurrence abilities, have a crucial role in VM.^84^

## Future settings in anti-angiogenic therapy

Before we endeavor for designing novel and effective strategies in anti-angiogenic therapy, it is important to prioritize the compensatory angiogenic mechanisms as targets for improving the therapeutic index of anti-angiogenic regime. Agents counteracting the redundant functioning of pro-angiogenic pathways and growth factors, patrolling the conspiracy of BMD tumor and stromal cells including pericytes and CSCs, antagonizing the angiogenesis-independent alternative mechanisms of vascular remodeling and overcoming the rebounds of invasiveness and metastasis after drug holidays should be an integral part of a judiciously formulated combinatorial therapeutic approach of future anti-angiogenic settings.^85^ Moreover, the systems pharmacology approaches integrating the biology of anti-VEGF resistance with the prognostic, predictive, pharmacodynamic and surrogate biomarkers coupled with computational imaging techniques such as dynamic contrast-enhanced magnetic resonance imaging and other imaging tools monitoring the vessel permeability, integrity and tumor perfusion might be helpful in ameliorating the drug resistance and optimizing the performance of anti-angiogenic drugs ^86^.

## Conclusion

Besides the emerging challenges, anti-angiogenic therapy has benefited not only for malignant diseases but also for non-malignant human ailments as well. Before launching of the anti-angiogenic therapy from the cradle to its action port, it was described as a therapy of ‘resistant to resistance,' owing to the genetic stability of ECs, as anti-angigenic drugs mostly target ECs.^2^ However, this turned out to be an illusion with growing resistance toward anti-angiogenic agents. Sizable literature has accumulated in the recent past describing the fact that tumors employ multiple mechanisms of vascularization that compensates the treatment of the currently used anti-angiogenic agents. In the midst of emerging resistance to the currently available anti-angiogenic agents, future settings should capitalize more on developing novel anti-angiogenic agents targeting compensatory angiogenic mechanisms, which may be used in combination with currently available anti-angiogenic agents. In the current situation, combination therapeutic regimes seems to be a possible approach for amelioration of tumor resistance, as several preclinical and clinical studies have demonstrated significant impact of combination therapy in improving the clinical benefits to the patients.
